# Is it possible to achieve an acceptable disease control by dietary therapy alone in Berardinelli Seip type 1? Experience from a case report

**DOI:** 10.3389/fendo.2023.1190363

**Published:** 2023-06-06

**Authors:** Carolina Cecchetti, Elisabetta Belardinelli, Paola Dionese, Rita Teglia, Roberta Fazzeri, M. Rosaria D’ Apice, Amanda Vestito, Uberto Pagotto, Alessandra Gambineri

**Affiliations:** ^1^ Division of Endocrinology and Diabetes Prevention and Care, Istituto di Ricovero e Cura a Carattere Scientifico (IRCCS), University Hospital of Bologna, Bologna, Italy; ^2^ Department of Medical and Surgical Sciences (DIMEC), Alma Mater Studiorum University of Bologna, Bologna, Italy; ^3^ Laboratory of Medical Genetics, Tor Vergata Hospital, Rome, Italy; ^4^ Gastroenterology Unit, Department of Digestive Diseases, S. Orsola-Malpighi Hospital, Bologna, Italy

**Keywords:** lipodystrophy, congenital generalized, hypolipaemic diet, AGPAT2 gene, metabolic complications

## Abstract

**Background and objective:**

Severe metabolic complications generally manifest at an early age in Berardinelli – Seip congenital lipodystrophy (BSCL) and their management is especially challenging. Nutritional intervention with low lipid diets is considered by experts to be fundamental in treating the disease when associated with medical therapy, however little is known about the beneficial effects of dietary interventions alone.

**Aim:**

To underline the importance of a well-structured low-fat diet in BSCL patients.

**Methods and results:**

A BSCL male patient strictly followed a hypocaloric hypolipemic diet (60% carbohydrates, 22% fats and 18% proteins) since clinical diagnosis at the age of one year. Interestingly, pharmacological interventions were not required at any point during the follow-up. Aged 16 years the patient was referred to our center. Biochemistry, hormonal evaluation, 75 mg oral glucose tolerance test, cardiac evaluation and abdominal ultrasound were performed, revealing no abnormalities. Genetic analysis and leptin dosage were carried out, confirming the diagnosis of BSCL type 1 (homozygosity for c.493-1G>C pathogenic variant in *AGPAT2* gene) and showing undetectable circulating levels of leptin (< 0.2 mcg/L). Diet therapy alone was therefore maintained, scheduling follow-up visits every six months, with acceptable disease control ever since.

**Conclusions:**

This report proves how a low-fat diet is of great help in the management of BSCL and its complications. In addition, a specific hypolipemic diet could be used alone as an effective treatment in selected cases with high compliance and, probably, a milder phenotype.

## Introduction

Congenital generalized lipodystrophy (CGL), also known as Berardinelli – Seip syndrome (BSCL), is an extremely rare genetic disorder (1–3).

The disease is suspected when a particular phenotype is present, characterized by near total loss of subcutaneous adipose tissue (SAT), which is responsible for lipid accumulation in ectopic sites, such as the liver and muscles. Consequently, severe metabolic complications are frequently present from a young age, with progressive development. Severe insulin resistance, type 2 diabetes, hypertriglyceridemia possibly causing acute pancreatitis, hepatic steatosis and hepatomegaly are the most common complications.

The prevalence of the disease varies among different ethnic groups; however it is estimated to be 0.96 in one million people in Europe (4), and approximately one in 10-12 million people worldwide (5).

CGL is an autosomal recessive disease, and its pathogenic mutations are known to affect adipogenesis and storage of triglycerides in adipocytes (6–9). The disease is classified into four different sub-types depending on the mutated gene: more specifically, CGL type 1 is caused by alterations located on 1 – acylglycerol-3-phosphate-O-acyltranspherase (*AGPAT2*) gene, encoding for the homonymous enzyme.

The phenotypical features are often present at birth, and the onset of metabolic abnormalities usually happens during infancy or adolescence (10, 11). Few cases of late diagnosis have been described. This is probably because CGL is an exceptionally rare disease and may go unrecognized in common clinical practice, but also because of its heterogenicity, sometimes presenting with milder phenotypes (12, 13).

We present the case of a 16-year-old male patient, who was referred to our center to confirm the diagnosis of BSCL clinically suspected at one year of age, and who had been treated with a hypocaloric hypolipidic diet since the clinical suspicion.

This report is particularly interesting because of the absence of metabolic complications and non-use of pharmacological treatment, thus highlighting the importance of nutritional intervention, especially in selected cases.

## Materials and methods

### Physical examination and biochemical assays

Physical examination included anthropometry (height, weight), blood pressure measurement, evaluation of pubertal stage and acanthosis nigricans. Height was measured without shoes and rounded to the nearest 0.5 cm; weight was measured without clothes. Body Mass Index (BMI) was calculated as weight (kg) divided by the square of height (m). Blood pressure was measured twice in the supine position, in the morning, after at least 3 min of rest before each measurement, taking the average of two. Pubertal stage was evaluated according to the Tanner staging system.

Blood sample measurements included hematology, metabolic parameters and hormones. Oral glucose tolerance test (OGTT) was performed with 75 g of glucose (Curvosio, Sclavo, Cinisello Balsamo, Italy).

Blood samples and tests were conducted in the morning after 12-h overnight fasting. Glucose, insulin, triglycerides, total and high-density lipoprotein (HDL) cholesterol, LH, FSH, oestradiol and testosterone were measured by Modular Analytics E170 (Roche Diagnostics, Mannheim, Germany) (14, 15). Low-density lipoprotein (LDL) cholesterol was calculated using the Friedewald formula (16). Insulin resistance was calculated using the homeostatic model assessment of insulin resistance (HOMA-IR) index (17).

Leptin was measured by ELISA from Mediagnost, Reutlingen, Germany.

MètaDieta^®^ software was used to calculate daily calorie consumption and percentages of fats, proteins, carbohydrates, fibers and micronutrients, with reference to the Italian food composition database.

### Dual-energy x-ray absorptiometry

Total body dual-energy x-ray absorptiometry (DEXA) was performed using a Lunar iDXA densitometer (DXA; GE Lunar iDXA, GE Healthcare, Bucks, UK).

### Liver elastography

Liver stiffness measurement (LSM) values were obtained using a FibroScan (Echosens, Paris, France) after an overnight fast, with prior complete abdominal ultrasound examination. LSM values were assessed as described in previous studies (18).

### Genetic analysis

Written consent was obtained from the patient and his parents.

NGS analysis was performed on genomic DNA extracted from peripheral whole blood sample using a Biorobot EZ1 automated system and the EZ1 DNA Blood Kit (Qiagen, Hilden, Germany). A custom-built NGS panel including the known genes causing congenital generalized or familial partial lipodystrophy (*AGPAT2* NM_006412. *BSCL2* NM_001122955, *CAV1* NM_001753, *PTRF* NM_012232, *LMNA* NM_170707, *PPARG* NM_015869, *PLIN1* NM_001145311, *CIDEC* NM_001199551, *LIPE* NM_005357, *AKT2* NM_001626) was used as described in Cecchetti et al. (19).

Sequencing data were processed with Torrent Suite Software (Thermo Fisher Scientific, Waltham, Massachusetts, USA). All the sequences were aligned with the Human Reference Genome assembly (GRCh37/hg19).

The calculated coverage of the coding sequence with a minimum depth of coverage of 50× was approximately 90.2%, with an exon padding of 25 bp. Regions not completely covered by NGS design, or which covered less than 50x were then analyzed by the Sanger sequencing method (primers available on request).

The variants were identified using Integrative Genomics Viewer software (IGV, Broad Institute, http://www.broadinstitute.org/igv/), and were annotated using the online Ion Reporter software (https://ionreporter.lifetechnologies.com/ir/secure/home.html).

All detected variants were classified based on database interpretation and literature data.

Sanger sequencing, using ABI 3500 Genetic Analyzer (Life Technologies), was used to confirm the candidate variants and for the subsequent segregation analyses.

## Case report

A 16-year-old male patient was referred to our endocrinology unit to genetically confirm the diagnosis of BSCL, which was clinically suspected at one year of age.

The patient was born at 34 weeks with a low birthweight (1610 gr) through elective caesarean section, due to intrauterine growth retardation.

Neonatal intensive care unit admission was required for 26 days. The patient was artificially breast-fed with weaning at 5-6 months of age: persistent growth difficulties were described.

Due to suspected muscular dystrophy, the patient was re-evaluated at one year of age.

**Figure 1:**
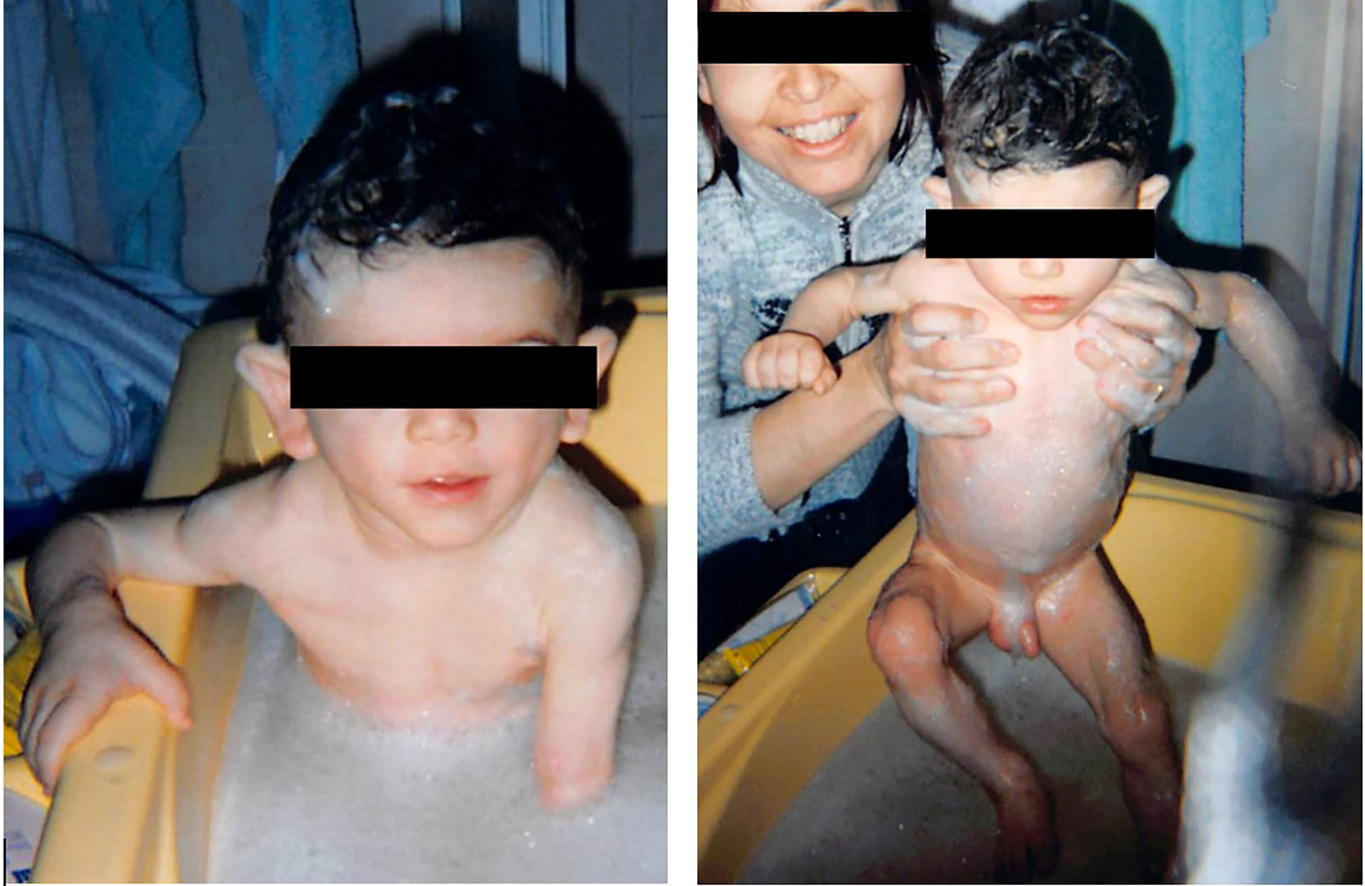
The patient at 1 year of age, showing a generalized lipodystrophic phenotype.

He was described as having a triangular face with prominent forehead, antimongoloid palpebral fissures and hollow cheeks (**Figure 1**). A pseudoathletic phenotype was also depicted, with muscular pseudohypertrophy and phlebomegaly, due to sub-total absence of SAT. Large hands and feet were also described. His mother stated that he had a voracious appetite, but an inability to achieve appropriate weight gain. Blood sample measurements were performed, revealing hypercholesterolemia (232 mg/dL; reference range 94–190 mg/dL) hypertriglyceridemia (129 mg/dL; RR 30-86 mg/dL) and hypertransaminasemia (Serum aspartate transaminase-AST 63 UI/L; RR 8-33 mg/dL). Consanguinity or similar phenotypes had not been reported in the family.

Based on the phenotypical abnormalities and metabolic alterations, a clinical diagnosis of BSCL was suspected. Genetic confirmation was not performed due to the unavailability of genetic screening.

Upon completion, a cardiac evaluation (electrocardiogram plus echocardiogram) and abdominal ultrasound were performed at the time of diagnosis, without detection of abnormalities. At one year of age, the patient was put on a hypolipidic diet, which was strictly followed over the years.

The patient was monitored yearly through blood analysis, cardiac evaluation, and abdominal ultrasound. No significant events were registered during follow-up, except for the detection of mild-moderate scoliosis and bilateral hydrocele (treated through ligation of patent processes vaginalis).

**Figure 2:**
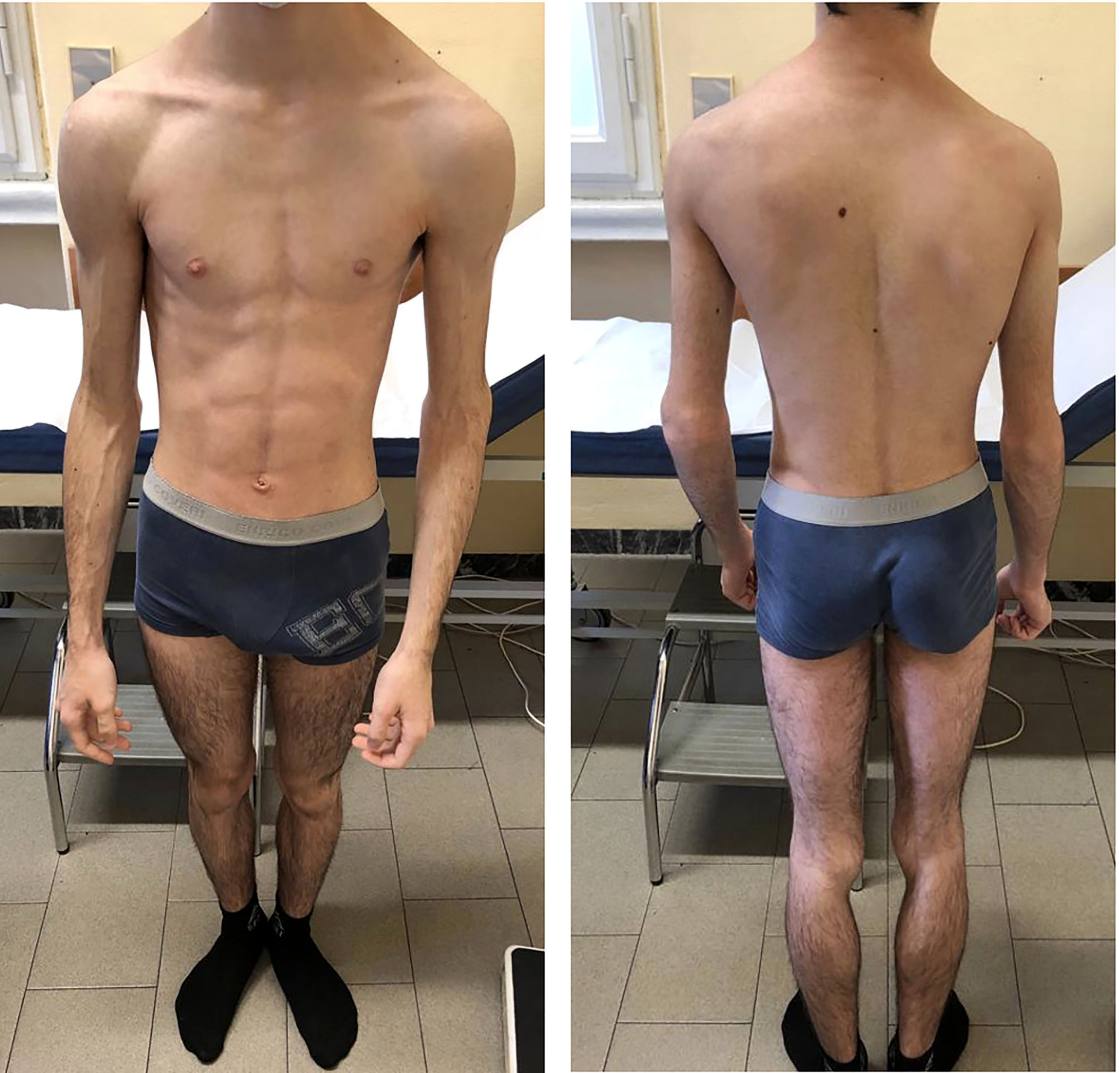
The patient at 16 years of age, at first evaluation at our Unit. Notable generalized lipodystrophy with upper limb phlebomegaly.

At our first physical evaluation, the patient presented a severe and generalized lack of SAT; phlebomegaly was also evident, particularly on the upper limbs (**Figure 2**). Acanthosis nigricans was not visible in any site examined. Pubertal stage was appropriate for the age. No organomegaly was detected.

Anthropometric data are described in **Table 1**. The patient’s BMI was 15.2 kg/m^2^.

**Table 1:** Patient general and anthropometric characteristics, biochemical and hormonal parameters at the referral time to our department (2021) and at last follow-up visit (2022).

	BSCL type 1 c.493-1G>C (2021)	BSCL type 1 c.493-1G>C (2022)	Reference Values
General and anthropometric characteristics			
Age, years	16	17	
*Height, cm*	162	162	
*Body weight, kg*	40	42.5	
Blood pressure, mmHg	135/75	120/70	< 130/80
**Tanner Stage**	P4/G4	P5/G4	
Biochemical parameters			
Serum creatinine, mg/dL	0.71	0.83	0.5 – 1.20
Basal glucose, mg/dL	95	71	60 – 110
Basal insulin, µU/mL	7.0	6.2	1.9 – 23.0
Homa- IR	1.62	1.09	< 2.5
Glycated hemoglobin, mmol/mol	30	30	20-42
Total cholesterol, mg/dL	141	118	<200
HDL cholesterol, mg/dL	36	34	<35
LDL cholesterol, mg/dL	91	70	<150
Triglycerides, mg/dL	156	69	<150
AST, U/L	30	30	<50
ALT, U/L	48	47	<50
yGT, U/L	25	28	<42
CPK, U/L	129	116	<170
Hormonal parameters			
TSH, µU/mL	2.01	1.66	0.25 – 4.50
FT4, pg/mL	9.1		5.5 -12.0
FT3, ng/mL	3.9		2.4 – 4.0
FSH, mU/L	4.2	4.7	1.3 – 19.3
LH, mU/L	1.6	2.1	1.2 – 8.6
Oestradiol, pg/mL	21	28	19 – 35
Testosterone, ng/mL	2.59	3.98	1.75 – 7.81
Leptin, µg/L	0.2		0.25-3.20 (BMI <20kg/m^2^)
OGTT (75 mg)			
Glucose 0	87		60-110
Glucose 30	109		
Glucose 60	132		
Glucose 90	102		
Glucose 120	108		
Insulin 0	10		
Insulin 30	57.9		
Insulin 60	108.5		
Insulin 90	70.3		
Insulin 120	93.8		

HOMA-IR, Homeostasis Model Assessment – Insulin Resistance; HDL, high-density lipoprotein; LDL, low-density lipoprotein; AST, aspartate transaminase; ALT, alanine aminotransferase; yGT, gamma-glutamyl transferase; CPK, creatine phosphokinase; TSH, thyroid stimulating hormone; FT4, free thyroxine; FT3, free triiodothyronine; FSH, follicle stimulating hormone; LH, luteinizing hormone; OGTT, oral glucose tolerance test.

Plicometry confirmed a diffuse lipodystrophy and total body DEXA results further proved the generalized absence of SAT, as shown in **Table 2**.

**Table 2:** Total body dual-energy x-ray absorptiometry (DEXA) results.

Region	*Area (cm^2^)*	*BMC (g)*	*BMD (g/cm^2^)*	*Fat (g)*	*FFM + BMC (g)*	*Total (g)*	*% Fat*
**Left arm**	106.29	63.4	0.597	187.5	1489.6	1677.1	11.2
**Right arm**	123.28	78.84	0.640	176.9	1668.0	1844.9	9.6
**Left leg**	276.20	277.45	1.005	693.2	5866.6	6559.8	10.6
**Right leg**	257.63	263.25	1.022	625.8	5793.0	6418.8	9.7
**Trunk**		243.87		2474.4	17346.0	19820.4	12.5
**Pelvis**	219.30	243.87	1.112				
**Cost. L**	106.69	78.96	0.740				
**Cost. R**	110.64	86.9	0.778				
**Spine T**	146.60	130.36	0.889				
**Spine L**	51.37	56.09	1.092				
**Subtotal**	1397.99	1278.37	0.914	3157.7	32163.2	36320.9	11.4
**Head**	214.95	437.17	2.034	864.5	2957.4	3821.9	22.6
**Total**	**1612.94**	**1715.53**	**1.064**	**5022.2**	**35120.6**	**40142.8**	**12.5**

The bold values represent the total of each column.

Interestingly, hyperphagia was not reported by the patient or his parents.

Regarding the patient’s family history, his father showed a lean phenotype (BMI 20.2 kg/m^2^) and was affected by mild dyslipidemia and autoimmune hypothyroidism. The patient’s mother was obese (BMI 30.4 kg/m^2^) and suffered from petit mal epilepsy.

Laboratory tests are presented in **Table 1**.

The overall metabolic profile was within the normal range, except for a slight elevation in triglycerides and alanine aminotransferase (ALT). Testosterone levels were in the lower limits of the reference range.

Lastly, standard 75 mg OGTT showed normal glucose tolerance, but mild hyperinsulinemia was present following stimulation.

At the time of evaluation, the patient was not taking any medication and was only following dietary suggestions.

Information regarding dietary habits was obtained through a self-reported 14 – day food diary. The average daily calorie intake was 1750 Kcal, represented by 60% carbohydrates, 22% fats and 18% proteins. Interestingly, the percentage of calories provided by lipids was within the lower limits of the reference range (20 to 35%). Specifically, 5.9% of the total daily calorie requirement was provided by saturated fat, in accordance with the Suggested Dietary Target (SDT: <10%). Polyunsaturated fatty acid (PUFA) consumption was within the reference daily intake (5.1% RDI: 5-10% of total daily kcal). Simple carbohydrates represented 18.9% of the total daily kcal intake, slightly above the SDT defined limit (< 15%). Protein intake was 1.93 g/kg, twice the RDI (0.93 g/kg). Daily consumption of fiber was 7.57 gr/1000 kcal (adequate intake (AI): 8.4 g/1000 kcal).

Serum leptin showed undetectable levels (**Table 1**), as expected for BSCL (1). NGS analysis was conducted, and the patient was found to carry the pathogenic variant c.493-1G>C in *AGPAT2* gene in a homozygous state (20, 21). NGS analysis also detected a heterozygous variant located in LIPE gene c.666G>C, leading to amino acid substitution p.Trp222Cys. This variant has never been reported in the GnomAD database, but was defined as probably benign by Varsome software. Segregation analysis detected the same *AGPAT2* variant in both parents in a heterozygous state.

Abdominal ultrasound with liver elastography showed a normal-sized liver, with regular margins and homogeneous echotexture, without signs of hepatic steatosis. All other abdominal organs were described as devoid of any abnormalities. Liver stiffness was assessed with a result of 5.3 KPa, thus defining a F0-F1 Metavir fibrosis score.

Echocardiography and ECG were performed, resulting in normal cardiac morphology and function: more specifically, left ventricle was not hypertrophic, with normal ejection fraction and without segmental hypokinesia (end-diastolic septum width: 0.8 cm; end – diastolic posterior wall width: 0.8 cm, ejection fraction: 60%). Cardiac valvulopathy was absent. At ECG, normal sinus rhythm was present, without signs of conduction defect.

Lumbar DEXA results indicated normal bone mass density for age (Z score 0.6; BMD 1.009 g/cm^2^).

Considering the satisfying metabolic control of the disease, diet therapy was maintained without pharmacological intervention and a follow-up plan was scheduled with visits every six months. The patient is now 17 years old and is still following a hypolipemic diet plan without any metabolic complications. Diet specifics have not changed since the first evaluation.

Knowing that the AGPAT2 mutation is associated with lytic lesions in long bones (1, 2), a bilateral X-ray of the tibia and fibula was recently carried out. As expected, asymptomatic polylobate lytic lesions were detected bilaterally in the tibia diaphysis and in the mid-distal 1/3 of the fibula. The patient was thus referred to a specialized orthopedist for a further evaluation and possible treatment.

## Discussion

This report presents the case of a young male patient with BSCL, who succeeded in achieving adequate control of the disease through strict adherence to a hypolipemic diet.

BSCL type 1 is caused by homozygous mutations affecting the *AGPAT2* gene, located on chromosome 9q34, encoding for the homonymous 1 – *AGPAT2* enzyme. The enzyme is a lysophosphatidic acid acyltransferase isoform of 278 amino acids highly expressed in adipose tissue, and is responsible for the transformation of lysophosphatidic acid into phosphatidic acid (PA) through acylation. This process is fundamental in the biosynthetic pathways of triglycerides and glycerophospholipids, since PA is a precursor of both phospholipids and diacylglycerol.

In the case described, the patient presented the homozygous mutation c.493-1G>C in the *AGPAT2* gene, which has also been described in the literature (20, 21). The genetic alteration substitutes G for C at position-1 of intron 3, located in a highly conserved 3’ splicing acceptor site. As a result, the mutation may be responsible for defective splicing with deletion of the entire exon 4. This alteration does not affect the *AGPAT2* mRNA reading frame, however the translated protein seems to be missing a pivotal segment for its correct functioning. More specifically, c.493-1G>C mutation could be responsible for PTEGR domain loss, which has been recognized as fundamental for acyltransferase activity.

However, how *AGPAT2* deficiency causes the syndrome is still unclear, but impaired triglyceride synthesis and storage, blocked differentiation of adipocytes from precursors and apoptosis/necrosis of adipocytes seem to be involved (22). As a result, patients affected by BSCL type 1 manifest almost a total absence of SAT associated with precocious metabolic complications and markedly low serum levels of adipose-derived hormones, namely leptin and adiponectin.

Akinci et al. (11) reported the median onset of hypertriglyceridemia and hepatic steatosis at 14 years and 16 years of age respectively, however cases of earlier development are fairly common (10, 23–28). Genotype – phenotype correlations have also been described: in BSCL type 1, females tend to develop diabetes and acanthosis nigricans more than males (29). The present case seems to be in accordance with this tendency, since the patient had no sign of acanthosis nigricans or any glycemic alteration.

BSCL patients commonly die prematurely and have a shorter life expectancy of 30+ years compared to healthy individuals (30). Experts thus suggest an intensive approach aimed at the prompt management of disease complications, including lifestyle changes and appropriate medical treatment (1, 2). Pharmacological therapy in CGL is aimed primarily at treating the severe metabolic alterations, following current guidelines for the specific complication. Recombinant human methionyl leptin (metreleptin) is currently the only treatment officially indicated for lipodystrophic patients. Metreleptin is approved as a replacement therapy in association with diet to prevent and manage the complications of leptin deficiency, such as hypertriglyceridemia, diabetes mellitus, and hepatic steatosis (31).

In CGL, studies have demonstrated the safety and effectiveness of metreleptin treatment, showing significant and sustained improvement in the overall metabolic profile (32). In addition, Cook and colleagues associated metreleptin therapy with a reduction in mortality risk in BSCL treated patients (33).

Despite its efficacy, metreleptin needs to be combined with an adequate diet plan for a satisfactory metabolic improvement.

Energy restriction and low-fat intake are pillars in the treatment of lipodystrophy. Some authors (1, 8) have indicated a restriction of total fat intake of between 20-30% of total dietary energy as an efficient tool to lower serum triglycerides. In murine *AGPAT2*−/− models, a reduction in dietary fat led to a marked amelioration of the triglyceride liver concentration and hepatic steatosis, suggesting how a large proportion of hepatic triglycerides are derived from dietary fats (34).

Only a few studies have been aimed at showing the specific response to dietary treatment in BSCL.

In a retrospective study conducted on 8 BSCL children, Papendieck et al. analyzed how the metabolic profile responded to a diet adjusted to the reference daily intake (RDI) with a restriction of fast-acting sugars, without any pharmacological therapy (35). Interestingly, a marked improvement was observed, with a reduction in triglyceride serum levels, insulin resistance (HOMA-IR), liver enzymes and hepatomegaly, in less than one year of follow-up. When the patient did not consistently adhere to the diet, a simultaneous increase in serum triglycerides occurred, followed over time by other metabolic parameters, therefore making triglycerides levels the most sensitive variable to diet transgression.

Montenegro et al. (27) described a case of a 4-month-old BSCL female with severe metabolic complications: the patient was given a norm caloric modified milky diet, with 30% lipids (medium chain triglycerides), 15% proteins and 53% carbohydrates, together with insulin therapy. After one month of treatment, a successful reduction in serum fasting triglycerides was obtained, together with a marked improvement in glucometabolic control which eventually led to a discontinuation of insulin treatment. At 20 months of age, diet therapy alone was still effective in managing the metabolic complications.

Another study analyzed the effects of different dietary regimens in CGL (36). A 36-year-old female was administered intensive insulin therapy plus dL-fenfluramine and four diet plans with different fat and energy contents. Compared to the other regimens, the results showed that a low – fat diet was more effective in the overall glycemic control.

The metabolic effect of a nutritional intervention combined with zinc oral supplementation was studied by de Medeiros Rocha D et al. (37). The dietary plan consisted in 55% carbohydrates, 30% fats and 15% proteins, with the consumption of whole-grain and low glycemic index food. Zinc supplementation was provided according to RDI. After three months, the nutritional intervention lowered the energy consumption and glycated hemoglobin. The role of zinc was probably related to an enhancement in insulin and leptin effectiveness, although the precise mechanism still needs to be clarified.

Despite these promising data, long-term adherence to a strict diet can be challenging, especially during developmental ages, where food restriction should be balanced with requirements needed for adequate growth (2). A previous study (36) showed that caloric restriction was responsible for undesirable weight loss and persistent hunger in a CGL patient, however the diet was not characterized by a low lipid intake. However, Papendieck et al. (35) showed a decrease in hunger when BSCL patients were rigorous in diet adherence, which was also observed in our case study.

This report underlines the fundamental role of diet in managing BSCL. From our experience, further lowering lipids to 20-25% of daily caloric intake could ameliorate metabolic complications, making their management easier for the clinician. Current dietary recommendations rely on clinical experience and scarce literature. Further research is crucial to be able to define precise nutritional interventions in these ultra-rare cases.

It is possible that BSCL manifests itself in milder phenotypes more than is commonly believed, thus challenging physicians in making an already difficult diagnosis. Studies on genotype – phenotype correlation in BSCL type 1 are scanty. Further studies could improve the understanding of the disease and help in tailoring the diagnostic and therapeutic protocol.

## Data availability statement

The datasets for this article are not publicly available due to concerns regarding participant/patient anonymity. Requests to access the datasets should be directed to the corresponding author.

## Ethics statement

Written informed consent was obtained from the individual(s), and minor(s)’ legal guardian/next of kin, for the publication of any potentially identifiable images or data included in this article.

## Author contributions

CC, PD, EB and AG collected clinical data and wrote the manuscript. RT and RF collected dietary data and contributed in writing the manuscript; MD performed the genetical analysis and revised the manuscript. AG and UP revised the manuscript. AV contributed efficiently in adding necessary information for manuscript correction and revision. She performed abdominal ultrasound and liver elastography in the described case. All authors contributed to the article and approved the submitted version.
